# Characteristics of ovarian cancer detection by a near-infrared fluorescent probe activated by human NAD(P)H: quinone oxidoreductase isozyme 1 (hNQO1)

**DOI:** 10.18632/oncotarget.18044

**Published:** 2017-05-20

**Authors:** Yuko Nakamura, Zhenhua Shen, Toshiko Harada, Tadanobu Nagaya, Kazuhide Sato, Shuhei Okuyama, Fusa Ogata, Peter L. Choyke, Robin L. McCarley, Hisataka Kobayashi

**Affiliations:** ^1^ Molecular Imaging Program, Center for Cancer Research, National Cancer Institute, United States National Institutes of Health, Bethesda, Maryland 20892-1088, USA; ^2^ Department of Chemistry, Louisiana State University, Baton Rouge, Louisiana 70803-1804, USA

**Keywords:** near-infrared emitting probe, human NAD(P)H: quinone oxidoreductase isozyme 1, green light-emitting probe, kinetic map, peritoneal cancer metastases

## Abstract

Near-infrared (NIR) fluorescent probes are ideal for *in vivo* imaging, because they offer deeper tissue penetration by the light and lower background autofluorescence than fluorophores that emit in the visible range. Q_3_STCy is a newly synthesized, NIR light-emitting probe that is activated by an enzyme commonly overexpressed in tumor cells, human nicotinamide adenine dinucleotide (phosphate): quinone oxidoreductase isozyme 1, known as hNQO1 or DT-diaphorase. The purpose of this study is to compare the sensitivity of detecting peritoneal ovarian cancer metastasis (POCM) with Q_3_STCy and gGlu-HMRG, a green fluorescent probe, upon their surface application. *In vitro* uptake of Q_3_STCy was significantly higher than that of gGlu-HMRG. Using a red fluorescence protein (RFP)-labeled *in vivo* tumor model of POCM, the Q_3_STCy probe provided high sensitivity (96.9%) but modest specificity (61.0%), most likely the result of albumin-probe interactions and non-specific activation in nearby altered but healthy cells. Three types of kinetic maps based on maximum fluorescence signal (MF), wash-in rate (WIR), and area under the curve (AUC) allowed for differentiation of the activated fluorescence signal associated with POCM from the background signal of the small intestine, thereby significantly improving the specificity of Q_3_STCy to 80%, 100%, and 100% for MF, WIR, and AUC, as well yielding a moderate improvement in sensitivity (100% for all approaches) that is comparable to that with gGlu-HMRG, but with the added advantages of NIR fluorescence as the transduction modality. Such a new methodology has the potential to afford identification of cancerous lesions deeper within tissue.

## INTRODUCTION

The success of oncologic procedures, such as surgery, depends on the rapid and accurate localization of cancers, followed by their complete resection or ablation. Although large tumors are visible to the unaided human eye, tiny foci (< 2 to 3 mm) of cancer may be impossible to see. Consequently, optical fluorescence-guided imaging is being investigated as a tool capable of augmenting the human eye so as to improve the success of oncologic resections.

There are several major categories of fluorescent probes designed for imaging applications, but the primary consideration is whether probe emission is ‘always-on’ or ‘activatable’ in nature [1]. Always-on probes for identification of target tissues have the advantage of simplicity in their design and synthesis, but the disadvantage of high background signal, with the latter leading to the requirement of considerable time to clear the always-on probe from nearby healthy tissue so as to achieve adequate target-to-background ratios (TBRs). On the other hand, activatable probes have much lower background signals, thereby greatly improving the TBR, but they must exhibit rapid activation for their implementation to be practical; this latter requirement is key to the future of non-systemically delivered activatable probes [2]. A particularly useful method of probe activation is related to up-regulated enzymatic activities associated with cancers, and this area of research has given rise to a new generation of enzyme-activatable optical probes [3].

Another category of optical imaging probes is that based on the energy of excitation and emission of the probe or its corresponding reporter, specifically, whether such energies are within or outside of the visible range. Selection of a given energy-range probe depends on the nature of the task at hand. For cancerous lesions located on the surface of tissues, use of visible light-based probes is very reasonable and has the advantage of not necessarily requiring additional equipment. However, for lesions at some depth from the surface, near-infrared (NIR) energies are preferred, due to their ability to offer deeper tissue penetration [1, 4, 5]. Depending on the dimensions and location of the cancerous tissue, the detection of intraperitoneal lesions may be achieved using green or NIR light-emitting probes. Green light-emitting probes have several advantages over their NIR light-emitting counterparts. For example, green light-emitting probes can usually be chemically designed to have a higher quantum yield and be of smaller molecular weight, with the latter being very important for guided surgery approaches employing topical (non-systemic) application of the probe. Additionally, green light-emitting probes can be seen with the naked eye. However, green light-emitting probes have disadvantages, including high absorption of light by tissue at the emission and excitation energies of the probe, as well as the challenge of distinguishing background tissue autofluorescence from fluorescence signal of the probe, as a result of their spectral overlap. These factors work to decrease the depth of tissue penetration and thereby limit the achievable TBR. NIR light-emitting fluorescent probes can visualize targets located deeper in tissues with superior TBR, but their use requires NIR cameras for visualization, as NIR-light is invisible to the naked eyes [4].

However, it is unclear whether there is an inherent advantage to NIR probes compared to visible light for tasks that might be required of an optical probe. In this study, we compare the *in vivo* fluorescence cancer imaging of a newly developed activatable NIR light-emitting probe, Q_3_STCy, activated by human nicotinamide adenine dinucleotide (phosphate): quinone oxidoreductase isozyme 1 (hNQO1) [6] with a previously described activatable green light-emitting probe, gGlu-HMRG, whose fluorescence is activated by *γ*-glutamyltranspeptidase (GGT). The GGT-based probe has previously been demonstrated to be capable of highly sensitive detection of peritoneal ovarian cancer metastases (POCM) in an animal model [7]. hNQO1 is a predominantly cytosolic enzyme that uses NADH or NADPH equally well as cofactors to specifically reduce quinones to hydroquinones [8]. The specific two-electron reduction of quinones to their hydroquinone form by hNQO1 is historically considered to be a detoxification mechanism, because this reaction bypasses the formation of the highly reactive semiquinone [9]. As a result, hNQO1 provides cells with multiple layers of protection against oxidative stress, including the direct detoxification of highly reactive quinones; the maintenance of lipid-soluble antioxidants in their reduced forms; and stabilization of the tumor suppressor p53 [10]. Chronic inflammation suppresses hNQO1 expression and may increase susceptibility to cell injury. Paradoxically, increasing evidence suggests that high expression levels of hNQO1 at the early stages of carcinogenesis may provide cancer cells with a growth advantage [11–13]. hNQO1 has also been reported to be overexpressed 2- to 50-fold in the cytosol of numerous human tumor cells (e.g., colon, breast, pancreas, lung, and ovary) [9, 14, 15]. Therefore, the Q_3_STCy probe has potential for aiding in the detection of tiny cancer foci, such as intraperitoneal metastases.

Herein, we compared Q_3_STCy, a NIR light-emitting probe activated by hNQO1, with gGlu-HMRG, a green light-emitting probe activated by GGT, to detect POCM in mice.

## RESULTS

### Synthesis and analysis of probe Q_3_STCy

#### Synthesis

The intermediate compound was first synthesized through coupling the quinone propionic acid to the linker 2-mercaptoethanol, based on a previously published method [16]. The resulting intermediate compound was then reacted with 14 equivalents of phosgene in anhydrous THF for 12 h at room temperature. After the remaining, unreacted phosgene was removed in vacuo, the crude product was dissolved in THF, and this was injected into a solution of TCy, which was first treated with sodium hydride in anhydrous THF for 20 min. After reaction overnight and vacuum removal of solvent, the final product Q_3_STCy was obtained using flashing column with methanol in dichloromethane as eluent. ESI-HRMS for [C_52_H_64_N_3_O_5_S]^+^: 842.4565 (observed); 842.4567 (calculated); 0.2 ppm error. ^1^H-NMR and ^13^C-NMR spectra are in the Supplementary Information, and they demonstrate the expected structural characteristics of Q_3_STCy.

#### Mechanism of activation

**Figure 1:**
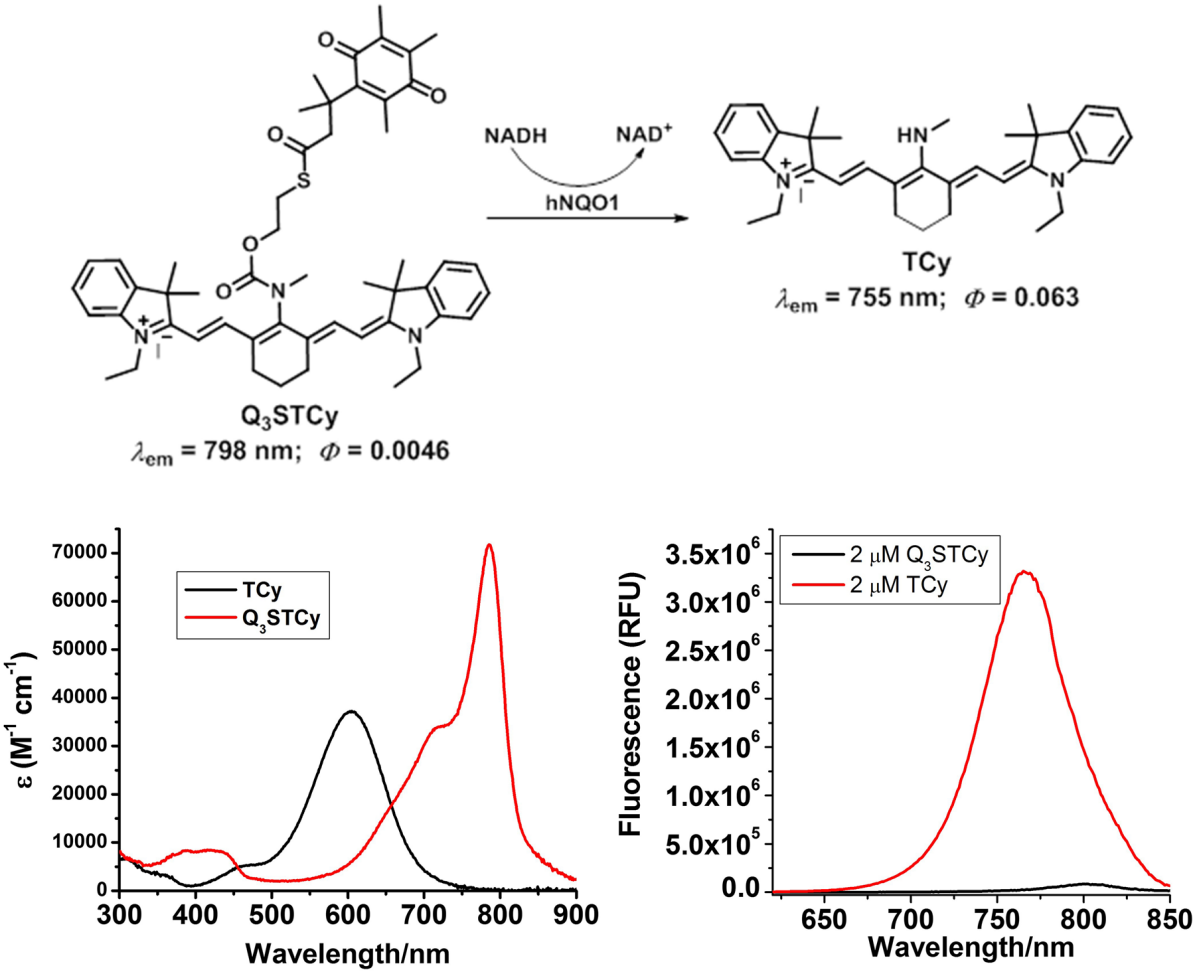
Chemical structure and their absorption and emission profiles of non-activated and activated Q_3_STCy (Q_3_STCy and TCy)

The fluorescence of Q_3_STCy is revealed by hNQO1 due to cleavage of the fluorescence quenching quinone propionic acid group upon its reduction by hNQO1 in the presence of NADH cofactor and subsequent self-cleavage of the 2-mercaptoethanol linker (Figure 1).

### *In vitro* fluorescence imaging

**Figure 2:**
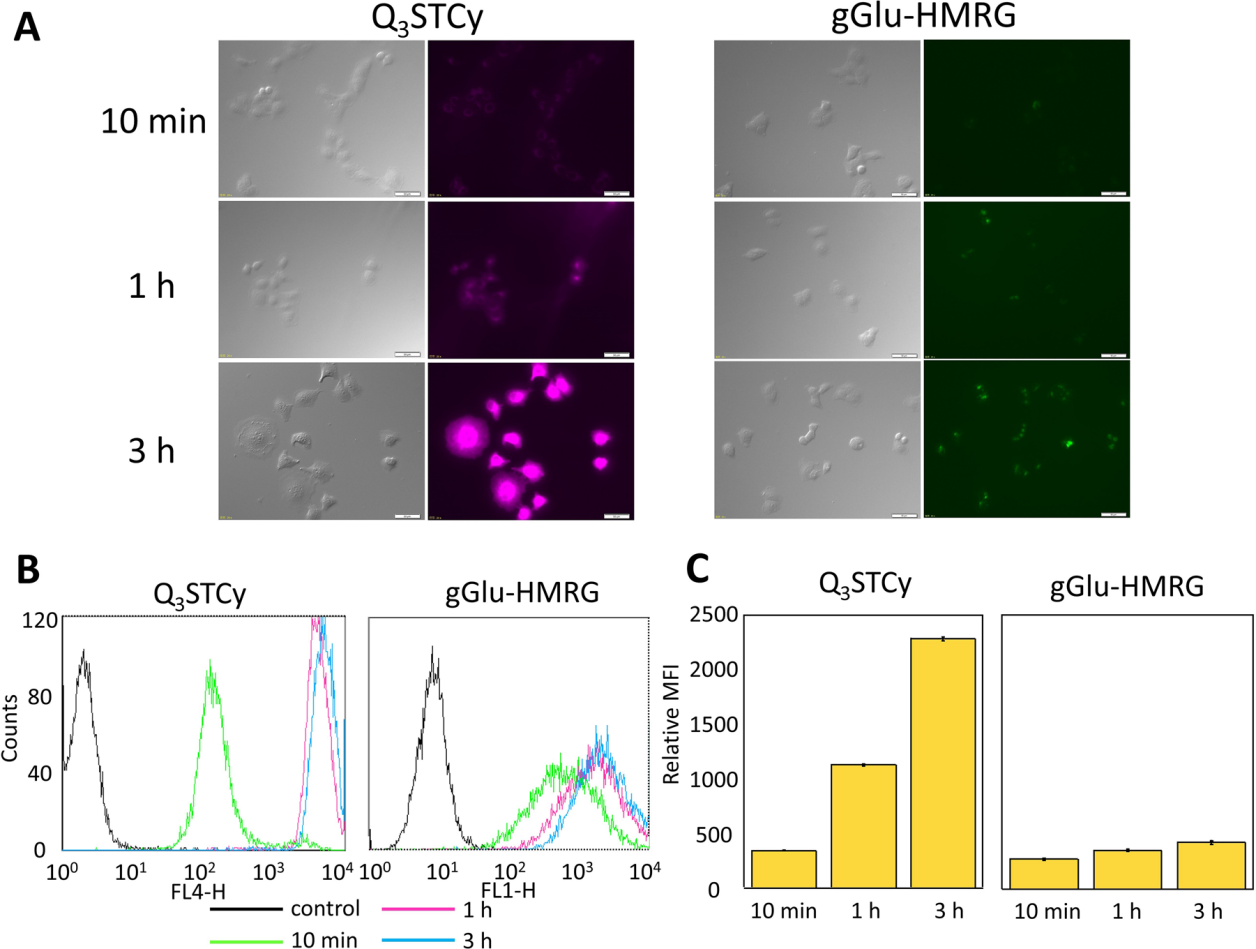
**(A)** Fluorescence microscopy studies. SHIN3 cells were incubated with Q_3_STCy and gGlu-HMRG for 10 min, 1, and 3 h. With 3 h incubation of Q_3_STCy SHIN3 cells showed stronger fluorescence compared to those incubated with gGlu-HMRG. **(B)** Flow cytometric analysis. One representative individual is shown. **(C)** Relative MFI of Q_3_STCy was significantly higher compared to that of gGlu-HMRG regardless of incubation time. Data are shown as mean relative MFI ± SEM.

SHIN3 cells showed weak activation and accumulation of Q_3_STCy or gGlu-HMRG probe after 10 min of incubation but strong activation and accumulation beyond 1 h incubation, as confirmed by fluorescence microscopy and flow cytometry (Figure 2). After 3 h incubation with Q_3_STCy, SHIN3 cells exhibited much more intense fluorescence compared to those incubated with gGlu-HMRG for the same amount of time. The relative MFI of Q_3_STCy was also significantly higher compared to that of gGlu-HMRG, regardless of incubation time (*p* < 0.01 for all incubation times) (Figure 2C).

### *In vivo* activatable imaging

**Figure 3:**
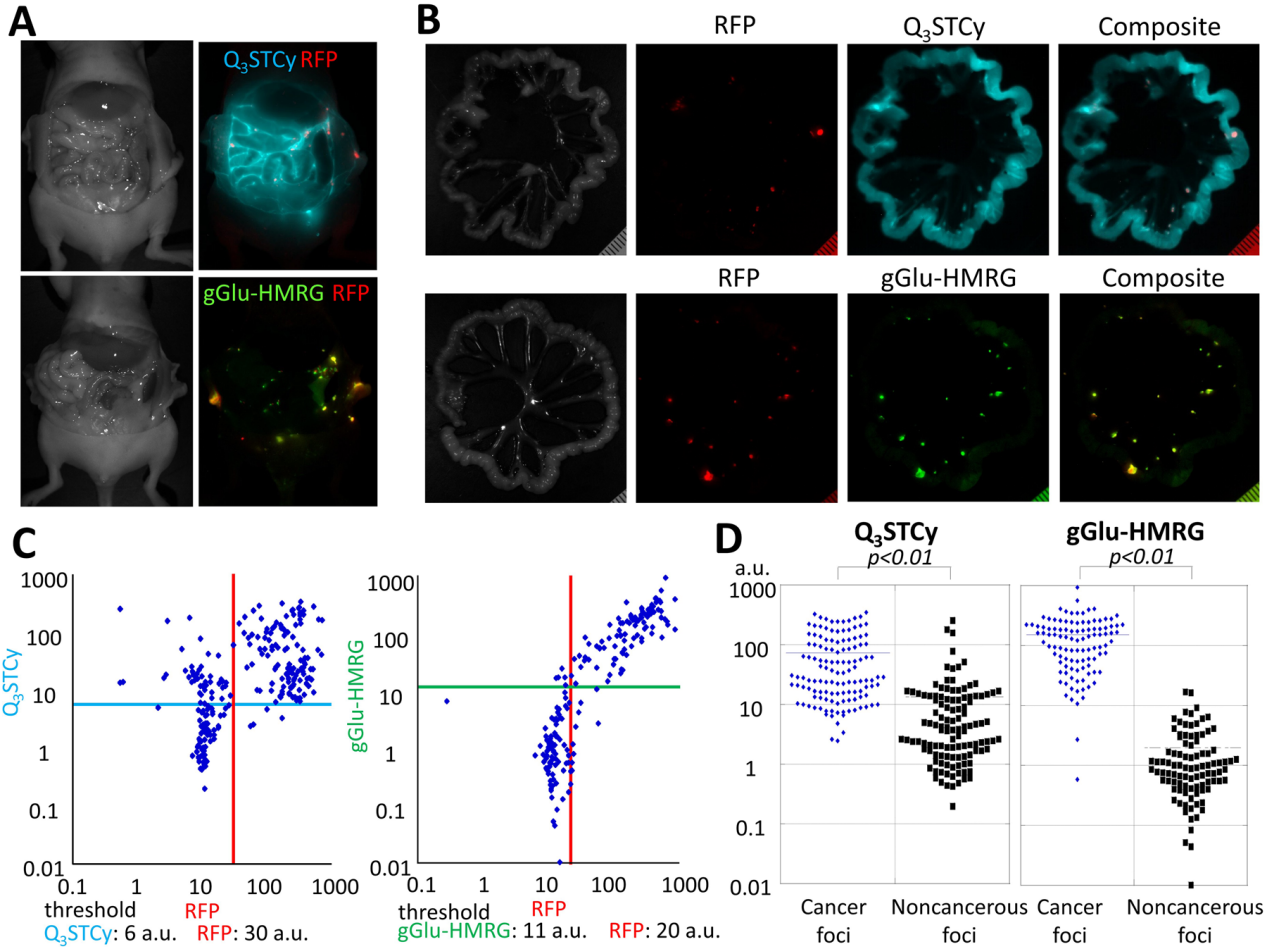
**(A)** Side-by-side images of RFP-transfected SHIN3 tumor-bearing mice injected i.p. with Q_3_STCy or gGlu-HMRG. The fluorescence signals of RFP (red), Q_3_STCy (light blue), and gGlu-HMRG (yellow green) were unmixed with their spectral library. **(B)** Side-by-side images of extracted mesenteries of with RFP-transfected SHIN3 foci and previously injected Q_3_STCy or gGlu-HMRG. Fluorescence signals of RFP (red) and Q_3_STCy (light blue), or gGlu-HMRG (yellow green) are mostly coincident. However, fluorescence signal of Q_3_STCy was also detected on surface of intraabdominal organ especially the small bowel. **(C)** Scatter plot of the fluorescence intensities in each nodule. When RFP was used as a reference for location of SHIN3 cells, the sensitivity and specificity of detecting SHIN3-RFP tumors were 96.9 and 61.0% for Q_3_STCy, and 97.1 and 97.6% for gGlu-HMRG, respectively. **(D)** Fluorescence intensity of cancer foci and noncancerous foci on the Q_3_STCy and gGlu-HMRG unmixed images.

*In vivo* imaging demonstrated that SHIN3-DsRed cells exposed to either Q_3_STCy or gGlu-HMRG exhibited distinct fluorescence signals that could be distinguished from the RFP fluorescence of SHIN3-DsRed cells. No detectable signals were observed in control animals that were not exposed to Q_3_STCy or gGlu-HMRG. After intraperitoneal injection of probes, the signals associated with fluorescence of gGlu-HMRG and Q_3_STCy were confirmed to be mostly coincident with RFP-positive foci. However, Q_3_STCy fluorescence was also detected on the surface of intra-abdominal organs, in particular the small bowel (Figure 3A). The side-by-side mesenteric images of the RFP-transfected SHIN3 animal models demonstrated that the fluorescence of Q_3_STCy and gGlu-HMRG were also mostly coincident with RFP-positive foci (Figure 3B), with fluorescence signal from the small bowel being most pronounced in the Q_3_STCy images. Using these images, the sensitivity and specificity of Q_3_STCy and gGlu-HMRG were calculated. 124 foci exhibited Q_3_STCy fluorescence among the 128 RFP-positive foci (96.9% sensitivity), while 46 foci possessed a fluorescence intensity > 6 a.u. among the 118 RFP-negative foci (61.0% specificity). 100 foci displayed gGlu-HMRG fluorescence above the threshold among the 103 RFP-positive foci (97.1% sensitivity), while two foci showed gGlu-HMRG fluorescence > 11 a.u. among the 84 RFP-negative foci (97.6% specificity) (Figure 3C).

From the Q_3_STCy unmixed image, a fluorescence intensity of 73.18 ± 82.95 a.u. and 13.48 ± 33.71 a.u. were found for cancer foci and noncancerous foci, respectively. The fluorescence intensity of cancer foci was significantly higher and statistically different than that of noncancerous foci (*p* < 0.01). Based on the gGlu-HMRG unmixed image, it was found that the fluorescence intensity was 152.68 ± 135.58 a.u. and 1.96 ± 2.92 a.u. for cancer foci and noncancerous foci, respectively. Once again, the fluorescence intensity of cancer foci was significantly higher than that of noncancerous foci (*p* < 0.01) (Figure 3D).

### *Ex vivo* activatable imaging

**Figure 4:**
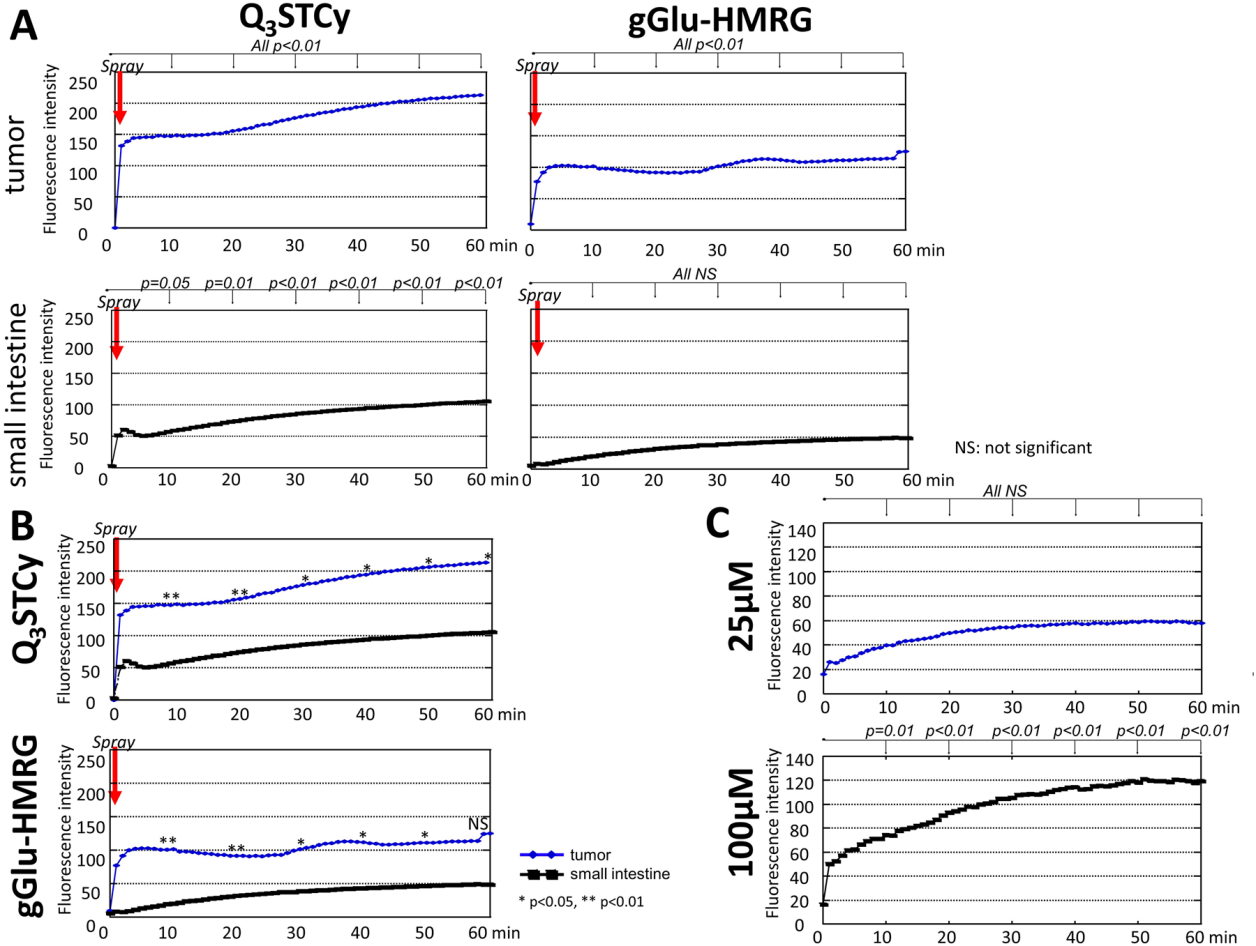
**(A)** Time fluorescence intensity curve of the tumor and small intestine after spraying Q_3_STCy and gGlu-HMRG. Difference at each time point compared to starting value was examined. **(B)** Time fluorescence intensity curve of the tumor and small intestine after spraying Q_3_STCy and gGlu-HMRG. Difference between tumor and small intestine was examined at each time point. **(C)** Time fluorescence intensity curve of Q_3_STCy after adding FBS. Difference at each time point compared to starting value was examined.

The intensity of fluorescence for extracted SHIN3 tumors increased significantly *immediately* after spraying the Q_3_STCy probe (*p* < 0.01 at all time points for probe-treated tumor vs prior to treatment) and gradually increased over the 60-min time period measured (Figure 4A and Supplementary Video 1). The fluorescence intensity of the healthy small intestine also increased in a statistically significant fashion upon its spray exposure to the Q_3_STCy probe, with a slight increase thereafter, up to 60 min (*p* = 0.05 and 0.01 at 10 and 20 min, *p* < 0.01 at 30, 40, 50, and 60 min after spraying the probe, respectively) (Figure 4A and Supplementary Video 1). *Importantly, the fluorescence intensity of the SHIN3 tumor exposed to the Q*_*3*_*STCy probe was higher than that of small intestine at all time points* (*p* < 0.01 at 10 and 20 min, and *p* = 0.01 at 30, 40, 50, and 60 min after spraying the probe, respectively) (Figure 4B).

Extracted SHIN3 tumors demonstrated an increase in fluorescence over the first several minutes after spraying of the gGlu-HMRG probe, followed by a relatively time-invariant signal (*p* < 0.01 at all time points) (Figure 4A and Supplementary Video 2). It was found the fluorescence intensity of the small intestine increased slightly but not significantly after application of the gGlu-HMRG (*p* = 0.54, 0.27, 0.15, 0.10, 0.07, and 0.08 at 10, 20, 30, 40, 50, and 60 min after spraying the probe, respectively). The measured fluorescence intensity of the SHIN3 tumor exposed to the gGlu-HMRG probe was higher than that of small intestine up to 50 min but not at 60 min (*p* < 0.01 at 10 and 20 min, *p* = 0.01, 0.02, 0.03, and 0.08 at 30, 40, 50, and 60 min after spraying the probe, respectively) (Figure 4B).

**Figure 5:**
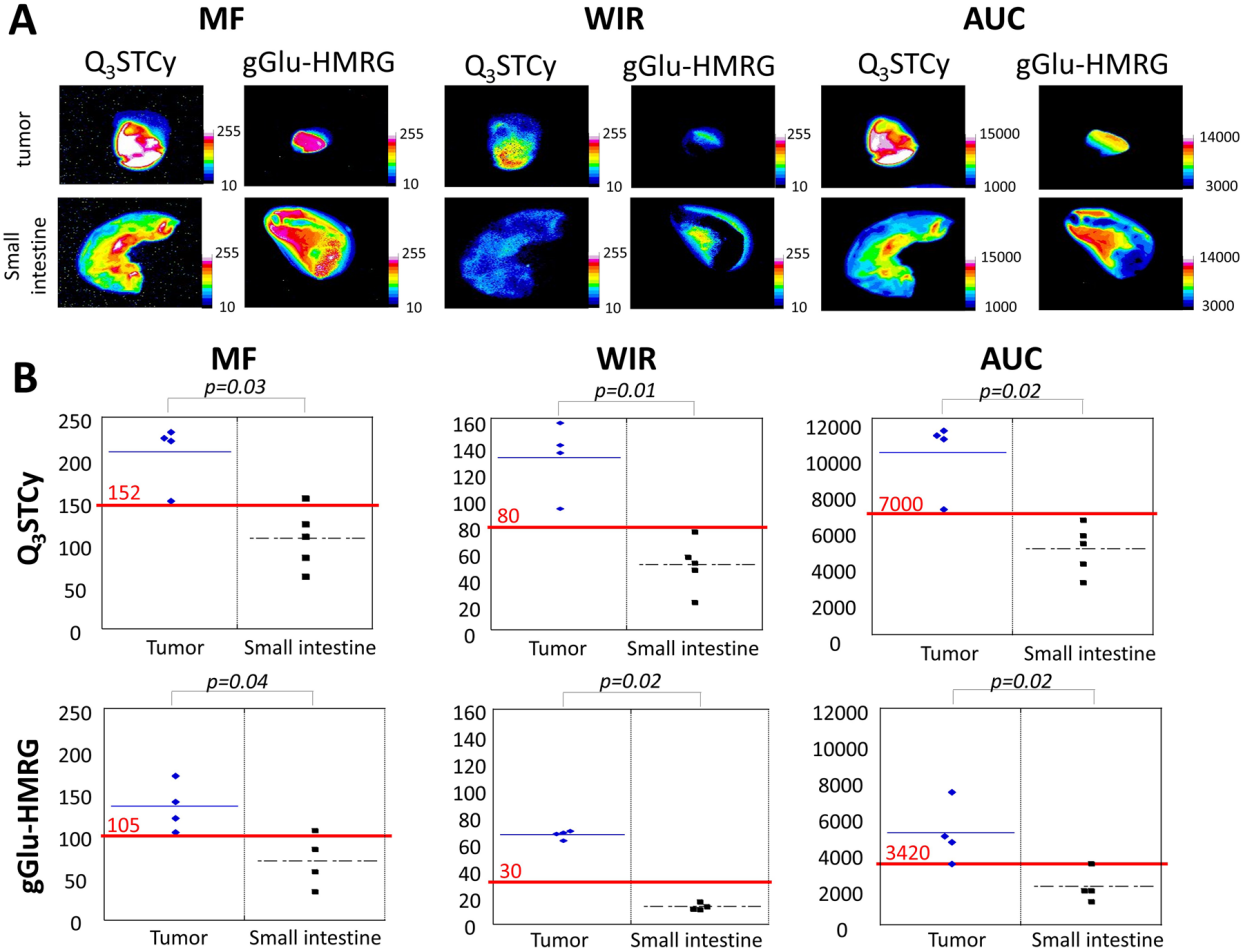
**(A)** Kinetic maps (MF, WIR, and AUC maps). On kinetic maps using both Q_3_STCy and gGlu-HMRG tumor showed clearly higher value compared to small intestine. **(B)** Three parameters derived from kinetic map of tumor and small intestine. Red value and line indicate the threshold value.

The MF, WIR, and AUC were determined to be significantly higher in the tumor versus the small intestine when using the NIR probe (*p* = 0.03, 0.02 and 0.01 for MF, WIR, and AUC, respectively). In addition, the MF, WIR, and AUC were also significantly higher in the tumor than the small intestine exposed to the gGlu-HMRG (*p* = 0.04, 0.02 and 0.02 for MF, WIR, and AUC, respectively) (Figure 5A and Table 1). The sensitivity and specificity of the Q_3_STCy probe, as determined by threshold values for diagnosing tumors, was 100% and 80%, 100% and 100%, and 100% and 100%, for MF, WIR, and AUC, respectively. The sensitivity and specificity of gGlu-HMRG based on threshold values was 100% and 75%, 100% and 100%, and 100% and 100%, for MF, WIR, and AUC, respectively (Figure 5B).

**Table 1:** Three parameters calculated from subtracted dynamic images

	Tumor	Small intestine	*P* value
Q_3_STCy			
MF	224.8 (81.2)	109.5 (92.1)	0.03
WIR	136.7 (64.9)	50.8 (34.1)	0.02
AUC	11004.5 (4352.0)	5073.4 (3454.8)	0.01
gGlu-HMRG			
MF	131.1 (66.1)	70.9 (72.3)	0.04
WIR	67.6 (7.1)	12.5 (6.0)	0.02
AUC	4809.1 (3947.1)	1905.0 (2112.1)	0.0

Data are the median (range).

### Dequenching of Q_3_STCy fluorescence

Q_3_STCy exhibited minimal fluorescence intensity before adding FBS. However, after adding FBS to 100 *μ*M Q_3_STCy, the fluorescence increased up to 60 min (*p* = 0.01 at 10 min, < 0.01 at 20, 30, 40, 50, and 60 min). When FBS was added to 25 *μ*M Q_3_STCy, the fluorescence increased gradually but not significantly (*p* = 0.30, 0.13, 0.08, 0.06, 0.06, and 0.06 at 10, 20, 30, 40, 50, and 60 min after adding Q_3_STCy, respectively) (Figure 4C). This increase in fluorescence is due to unfolding of the quenched Q_3_STCy probe, not interferent-induced activation/removal of the quinone group of the probe, as verified by mass spectrometry inspection of such solutions (Supplementary Information). Thus, the probe is not activated by the presence of FBS, but rather its ∼820-nm emission is increased, most likely the result of decreased PeT quenching between the Q_3_ functionality and the Cy group.

## DISCUSSION

The *in vitro* study using SHIN3 cells in monolayer culture demonstrated weak activation of Q_3_STCy and gGlu-HMRG probe and intracellular accumulation of their respective reporters after a 10-min incubation, but strong activation and accumulation was observed when the SHIN3 cells were exposed to the probes for times greater than 1 h, especially in the case of Q_3_STCy. In addition, the relative MFI found for Q_3_STCy was significantly higher compared to that of gGlu-HMRG, regardless of incubation time (10 min, 1 and 3 h). Thus, we conclude that Q_3_STCy is a promising activatable NIR probe for detecting POCM, with its capabilities being comparable to the previously established, excellent activatable probe, gGlu-HMRG.

Q_3_STCy demonstrated very high sensitivity for POCM. However, fluorescence was also detected on the surface of intraabdominal organs, especially the small bowel. This greatly reduced the specificity of the agent (61.0%) in the abdomen, a significant concern, as POCM are often found in association with the small bowel. These results revealed that Q_3_STCy can detect POCM with very high sensitivity, but modest specificity, most likely the result of probe activation in nearby healthy cells possessing higher-than-normal hNQO1 activity that results from their being *stimulated* by products released from their cancer cell neighbors [13, 17, 18]. Despite this limitation, activation of Q_3_STCy by cancer foci was significantly higher than by noncancerous tissue.

To further augment the capabilities of the new NIR probe so as to identify tumor-specific fluorescence signal, we employed kinetic parameters of the time-dependent activated Q_3_STCy fluorescence signal to distinguish cancerous and noncancerous tissue [19]. *Ex vivo* activatable imaging showed that the fluorescence intensity of SHIN3 tumors exposed to the NIR probe was higher and developed more rapidly than that of small intestine tissue exposed to the Q_3_STCy probe at all time points. Moreover, the MF, WIR, and AUC kinetic parameters with Q_3_STCy were significantly higher in tumors versus small intestine tissue. Using WIR and AUC as the criteria, the sensitivity and specificity based on the threshold values for diagnosing tumors was determined to be 100 and 100%. Thus, with these findings in hand, we anticipated that the non-specific background signal of Q_3_STCy in tissues can be overcome by kinetic maps derived from dynamic fluorescence imaging during *in vivo* studies of POCM.

gGlu-HMRG was used as a reference compound in this study. It demonstrated very high sensitivity and specificity for detecting POCM (97.1% and 97.6%, respectively), without using kinetic maps. However, such a green light-emitting probe has some potential disadvantages in clinical situations, including light absorption by tissue and background autofluorescence [2]. One approach to reduce autofluorescence is to unmix known autofluorescence spectra from the optical probe spectra. Indeed, we performed ‘unmixing’ to reduce the autofluorescence from small intestine. However, current spectral imaging is time-consuming, as it requires at least several seconds per frame; thus, this method is not necessarily well matched to real-time imaging applied to surgical or endoscopic procedures [1, 20]. gGlu-HMRG has been reported to be able to detect intraperitoneal metastases in preclinical mouse models rapidly (within 10 min) because of its rapid and strong activation upon contact with GGT on the surface of cancer cells; however, concern has been expressed about the limited penetration distance of green light in human-scaled applications [7].

Fluorescent probes that absorb and emit light in the NIR region are attractive because of their low phototoxicity, and minimal absorption of associated excitation/emission light by tissue and low autofluorescence background due to the energy of the light. As a result of these characteristics, NIR probes afford the opportunity to detect biological events deep within tissues. Indeed, autofluorescence from the small intestine did not hamper the detection of activated Q_3_STCy fluorescence signal from POCM, and such successful detection of cancerous tissue did not require time-consuming spectral unmixing. Moreover, tiny peritoneal tumors may hide in the folded mesentery because it may be difficult to stretch the whole mesentery and folded tissue is inevitable. Q_3_STCy, a NIR light-emitting probe, may therefore, be useful in such applications despite the need for a special camera [4].

Genetic reporters have been reported to illuminate cancer selectively *in vivo* [21–28]. However, ethical considerations are needed clinically, because genetic reporters require virus administration. In addition, genetically engineered mouse models of cancer reproduce the tumor microenvironment, including the intact immune response, more accurately than human cell line xenograft models [29, 30]. Yet, complicated techniques are required to develop genetically engineered mouse models. Thus, we chose a simple intraperitoneal xenograft tumor model.

In conclusion, we describe a new activatable probe, Q_3_STCy, whose hNQO1 activation leads to emission of NIR light. Q_3_STCy showed high sensitivity but modest specificity for detecting POCM. However, this modest specificity could be overcome by creating kinetic maps. The advantages of activatable NIR light-emitting probes in POCM include deeper tissue penetration and less background interference from autofluorescence. Thus, we suggest that Q_3_STCy is a new alternative to existing activatable probes for POCM which performs at very high sensitivity and specificity when kinetic parameters are considered.

## MATERIALS AND METHODS

### Synthesis and analysis of probe Q_3_STCy

Q_3_STCy was synthesized as described previously [6]. Quinone propionic acid [31] and the tricarbocyanine fluorophore TCy [32] were synthesized using standard procedures. All of the starting chemicals were purchased from Sigma-Aldrich or Fisher Scientific and used as received. Crude products were purified with 10-g silica columns (Biotage) using methanol/dichloromethane eluent. The Q_3_STCy probe was characterized by ^1^H-NMR, ^13^C-NMR, and ESI-HRMS.

### Cell lines and culture

The established ovarian cancer cell line, SHIN3 was used for *in vitro* fluorescence microscopy and flow cytometry. SHIN3-DsRed, in which the red fluorescent protein (RFP DsRed2)-expressing plasmid (Clontech Laboratories, Mountain View, CA, USA) was previously transfected, was used as the animal model of POCM [33]. Cell lines were grown in RPMI 1640 supplemented with 10% FBS and 1% penicillin-streptomycin (Life Technologies) in tissue culture flasks in a humidified incubator at 37°C in an atmosphere of 95% air and 5% carbon dioxide.

### *In vitro* fluorescence microscopy and flow cytometry

To compare fluorescence intensities of Q_3_STCy activated by hNQO1 and gGlu-HMRG activated by GGT, we performed fluorescence microscopy with SHIN3 cells. 4 × 10^4^ cells from each cell line were plated on a culture well covered by a glass cover slip and incubated in culture media for 24 h. Q_3_STCy or gGlu-HMRG (2 *μ*M) was added to the culture medium and incubated for 10 min, 1, and 3 h. After incubation, cells were washed once with phosphate-buffered saline solution (PBS), and fluorescence microscopy was performed using an Olympus BX61 microscope (Olympus America, Inc., Melville, NY) equipped with the following filters: excitation wavelength range 672.5–747.5 nm and emission wavelength range 765–855 nm for Q_3_STCy and excitation wavelength range 450–490 nm and emission wavelength range 500–550 nm for gGlu-HMRG. Transmitted light differential interference contrast (DIC) images were obtained at the same time.

For flow cytometry, 1 × 10^5^ cells from each cell line were plated in a 24-chamber culture well and incubated for 24 h. Q_3_STCy or gGlu-HMRG (2 *μ*M) was added to the culture medium, and cells were incubated for 10 min, 1, and 3 h. A 488-nm argon ion laser was used for excitation. Signals from cells were collected with a 515 to 545 nm band-pass filter for gGlu-HMRG and a 653 to 669 nm band-pass filter for Q_3_STCy. Cells were analyzed using a FACS Calibur (BD BioSciences, San Jose, CA, USA). Relative mean fluorescence intensity (MFI) was quantified as the ratio MFI_target_ to MFI_control_ using CellQuest software (BD BioScience). Samples were assayed three times in duplicate.

### Animal model

All procedures were performed in compliance with the Guide for the Care and Use of Laboratory Animals [34] and approved by the local Animal Care and Use Committee in NCI/NIH. Six- to 8-week old female homozygote athymic nude mice were purchased from Charles River (National Cancer Institute, Frederick, MD).

Peritoneal metastases in nude mice were established by intraperitoneal (i.p.) injection of 2 × 10^6^ SHIN3-DsRed cells suspended in 200 to 300 *μ*l of PBS. Studies were conducted 14–21 days after injection of the cells.

For an animal model of subcutaneous tumor, a subcutaneous injection of 2 × 10^6^ SHIN3 cells suspended in 200 *μ*l of PBS was performed in the right and left dorsi of select mice. The mice were evaluated 7–10 days after injection of the cells.

### *In vivo* activatable imaging

To examine the sensitivity and specificity of Q_3_STCy and gGlu-HMRG for imaging POCM, dilute aqueous solutions of Q_3_STCy or gGlu-HMRG (300 *μ*l of 25 *μ*M) were injected into the peritoneal cavities of RFP-transfected SHIN3 tumor bearing mice (*n* ≧ 4 for each group). Mice without injection of the probe under investigation were also examined as controls. Mice were euthanized with carbon dioxide 10 minutes after i.p. injection of each probe. After euthanasia, the abdominal walls were exposed, and mice were placed face up on a nonfluorescent plate. Whole abdominal images, as well as close-up images of the small bowel mesentery were obtained. Subsequently, the small bowel mesentery of each mouse was extracted and spread out on the nonfluorescent plate. Spectral fluorescence images were acquired using the Maestro *In-Vivo* Imaging System (Cri, Woburn, MA, USA). The following filter set was used for imaging RFP: a band-pass filter from 503 to 555 nm for excitation and a long-pass filter for emitted light above 645 nm; for imaging Q_3_STCy: a band-pass filter from 710 to 760 nm for excitation light and a long-pass filter for emitted light over 800 nm; and for imaging gGlu-HMRG: a band-pass filter from 445 to 490 nm for excitation light and a long-pass filter for emitted light over 515 nm. The tunable emission filter was automatically stepped in 10 nm increments from 600 to 800 nm for RFP, from 700 to 900 nm for Q_3_STCy, and from 500 to 600 nm for gGlu-HMRG, at constant exposure. The spectral fluorescence images consisting of spectra from autofluorescence, RFP, and Q_3_STCy or gGlu-HMRG were then unmixed, based on their spectral patterns, using commercial software (Maestro software; CRi).

For image analysis of Q_3_STCy, regions of interest (ROIs) were drawn within the tumor nodules depicted by the RFP images (cancer foci: average fluorescence intensity (AFI) ≧ 30 arbitrary units (a.u.)) and in the surrounding adjacent areas (noncancerous foci: AFI < 30 a.u.), and then the average fluorescence intensity of each ROI was calculated on the Q_3_STCy images with Maestro software. The number of ROIs drawn in the noncancerous areas was almost equal to the number drawn on cancer foci (*n* = 123). All visible nodules with areas > 0.05 mm^2^ on RFP images were analyzed. Additional ROIs were drawn on the nodules depicted on the Q_3_STCy images and compared to the RFP images to validate the existence of SHIN3 tumors. Positive signal for Q_3_STCy was defined as an AFI greater than 6 a.u.; a negative signal was defined as an AFI less than 6 a.u. The number of foci positive for both Q_3_STCy and RFP, and positive only for either Q_3_STCy or RFP was determined. The sensitivity of Q_3_STCy for the detection of POCM was defined as the number of peritoneal foci that were positive for both Q_3_STCy and RFP divided by the total number of peritoneal foci positive for RFP. Specificity of Q_3_STCy was defined as the number of peritoneal foci negative for both RFP and Q_3_STCy divided by the total number of peritoneal foci negative for RFP.

Image analysis of gGlu-HMRG was performed in exactly the same manner as that described for Q_3_STCy, except that the threshold for RFP was 20 a.u. and for gGlu-HMRG was 11 a.u.

The difference in fluorescence intensity on the Q_3_STCy or gGlu-HMRG images between cancer foci and noncancerous foci was also determined.

### *Ex vivo* activatable imaging

Kinetic maps derived from dynamic fluorescence imaging have been reported to be useful for overcoming the problem of dilution when using a sprayable or surface-applied optical probe, such as the two described here [19]. Thus, for differentiation between the fluorescence signal arising from specific and non-specific activation, we created kinetic maps using extracted subcutaneous SHIN3 tumor and small bowel, because the high fluorescence signal from the small bowel often hampers the detection of intraperitoneal metastases. Serial spectral fluorescence images were acquired using the Maestro *In-Vivo* Imaging System before and after spraying 10 *μ*l of 100 *μ*M Q_3_STCy or gGlu-HMRG solution every 1 min, up to 60 min after spraying the probes. The filter set and scanning parameters used for Q_3_STCy and gGlu-HMRG were the same as in the *in vivo* activatable imaging experiments. The spectral fluorescence images, consisting of spectra from autofluorescence, Q_3_STCy, or gGlu-HMRG were then unmixed, based on their spectral patterns using commercial software (Maestro software; CRi). Each ROI was drawn either within the tumor nodule or small intestine on the Q_3_STCy or gGlu-HMRG unmixed image and average fluorescence signal intensity was calculated.

Next, we calculated three previously reported kinetic parameters—maximum fluorescence signal (MF), wash-in rate (WIR), and area under the curve (AUC)—and then created kinetic maps based on these three parameters [19]. All images were analyzed using Image J software (http://rsb.info.nih.gov/ij/). First, ROIs were drawn on serial unmixed fluorescence dynamic images within the tumor and in the normal small bowel, and then the AFI of each ROI was calculated. Next, we generated a fluorescence intensity curve using a time series of images. Subtracted images were created by subtracting the pre images (initial images before spraying the probe) from each of the post images (images obtained after spraying the probe). Then, we calculated the following three parameters from each fluorescence intensity curve using subtracted images: MF, WIR, and AUC. MF is the maximum fluorescence signal observed during the entire dynamic image set. WIR (fluorescence intensity/ min) is the maximum slope approaching the MF. AUC is the area measured under the time–fluorescence curve. Kinetic maps based on these three parameters were created. Finally, we defined a threshold value for each parameter (152, 80, and 7000 for MF, WIR, and AUC with Q_3_STCy; and 105, 30, and 3420 for MF, WIR, and AUC with gGlu-HMRG, respectively). Using these threshold values, we calculated the sensitivity and specificity for diagnosing the presence of a tumor.

### Potential fluorescence dequenching of Q_3_STCy

To assess if changes in the environment of Q_3_STCy, result in an increase in its dequenched fluorescent state (probe unfolding), serial spectral fluorescence images were acquired using the Maestro *In-Vivo* Imaging System for 300 *μ*l of 25 *μ*M or 100 *μ*M Q_3_STCy before and after adding 300 *μ*l of FBS every 1 min up to 60 min. The tunable emission filter was automatically stepped in 10 nm increments from 700 to 900 nm at constant exposure. The spectral fluorescence images were then unmixed, using commercial software (Maestro software; CRi). Each ROI was drawn to encompass fluorescent signal in the a unmixed image, and the average fluorescence signal intensity was calculated using Image J software (http://rsb.info.nih.gov/ij/).

### Statistical analyses

Statistical analyses were performed with JMP 10 software (SAS Institute, Cary, NC). The difference of relative MFI between Q_3_STCy and gGlu-HMRG was determined with two-sided Mann–Whitney’s *U* test. We performed receiver-operating characteristic analysis to determine each threshold value. A two-sided Student’s *t*-test was used to compare the difference of fluorescence intensity between cancer foci and noncancerous foci. With respect to examining for non-specific activation of Q_3_STCy in the *ex vivo* activatable imaging experiments, we determined the differences in fluorescence intensity every 10 min after spraying probe compared to the starting value using Dunnett’s multiple comparison. The difference in fluorescence intensity and three parameters calculated from the subtracted dynamic images from tumor and small intestine were examined with a two-sided Mann–Whitney’s *U* test. Differences of *p* < 0.05 were considered statistically significant.

## SUPPLEMENTARY MATERIALS VIDEOS






