# Supplementary material for: Characteristics of ovarian cancer detection by a near-infrared fluorescent probe activated by human NAD(P)H: quinone oxidoreductase isozyme 1 (hNQO1)
Source: Oncotarget. 2017 May 20;8(37):61181–92. doi: 10.18632/oncotarget.18044 (PMC5617415; doi:10.18632/oncotarget.18044)
Supplement: Supplementary file 1 [file oncotarget-08-61181-s001.pdf]

Characteristics of ovarian cancer detection by a near-infrared fluorescent probe activated by human NAD(P)H: quinone oxidoreductase isozyme 1 (hNQO1)

SUPPLEMENTARY INFORMATION

SPECTRAL CHARACTERIZATION OF Q_3STCY PROBE

The ^{1}H-NMR and ^{13}C-NMR spectra of the probe are displayed below.

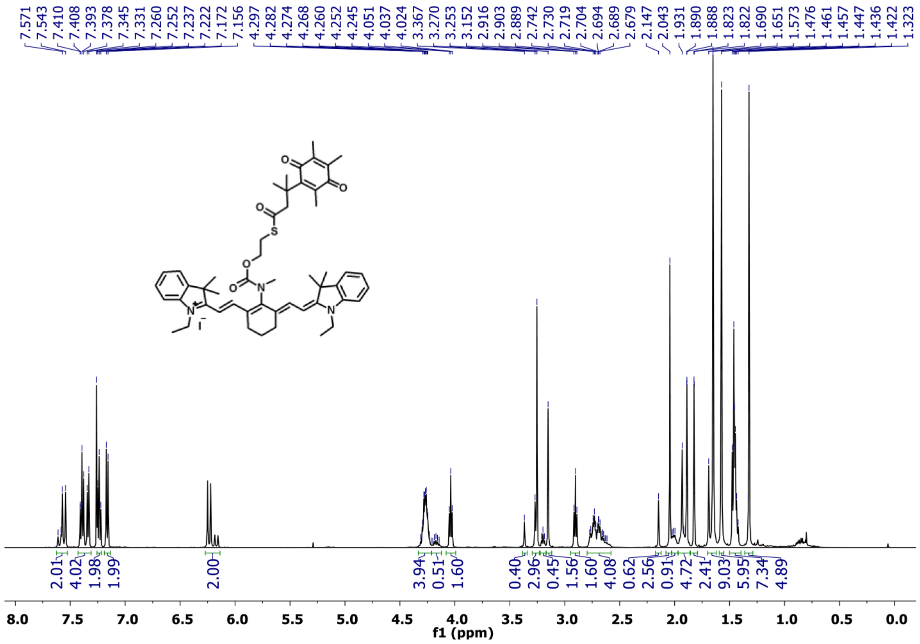

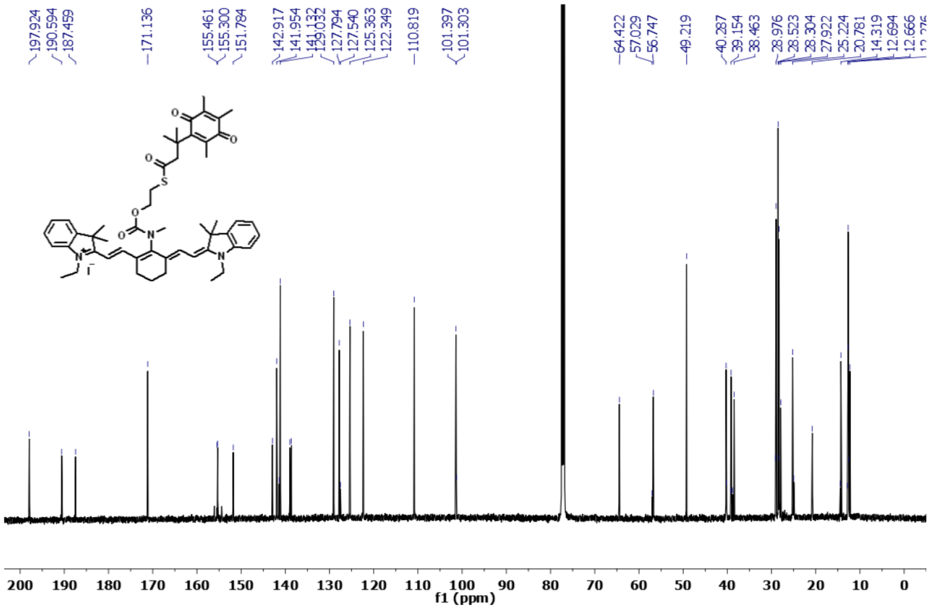

MASS SPECTRAL EVALUATION OF Q_3STCY PROBE EXPOSED TO FBS

25 μM Q_3STCy in PBS (2 ml) was mixed with 100% FBS (2 ml) to yield a 12.5 μM Q_3STCy solution, which was incubated for 1 h at room temperature prior to its examination by electrospray ionization-mass spectrometry. Besides ions associated with proteins at $m/z > 1000$, the only signal observed was for that of the Q_3STCy probe at $m/z = 842.4534$; there was no evidence to support the formation of the TCy reporter, having an $m/z = 506.3535$.

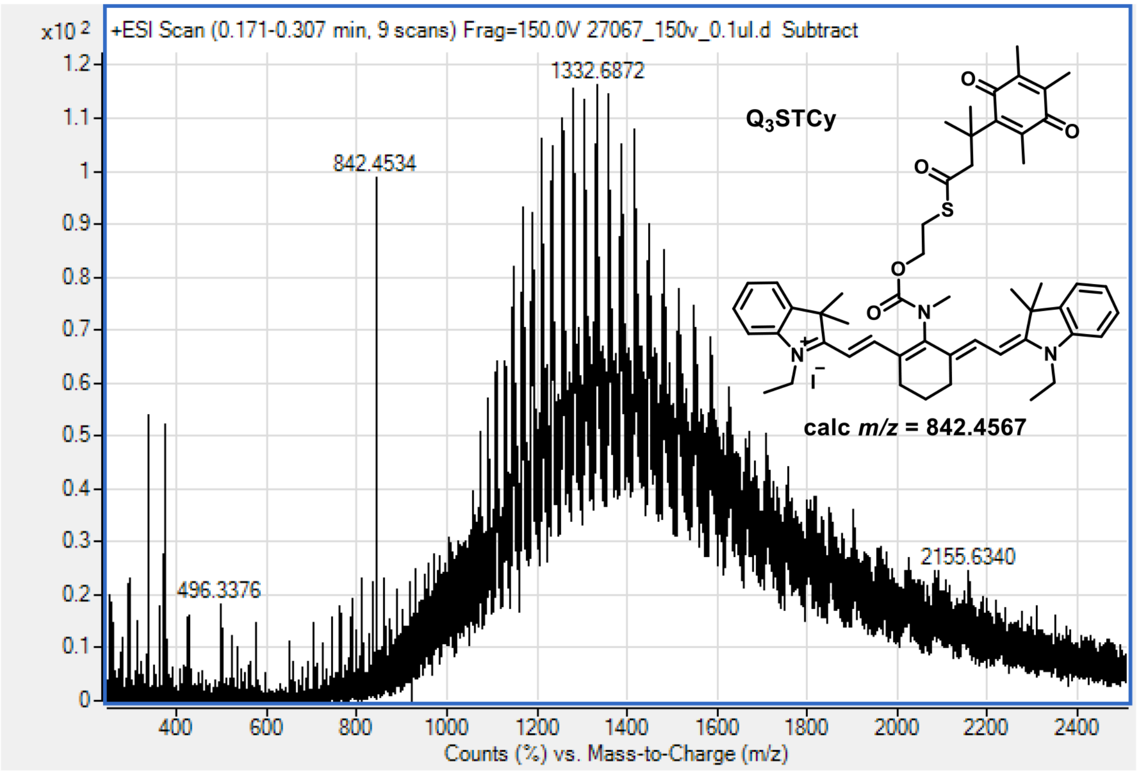

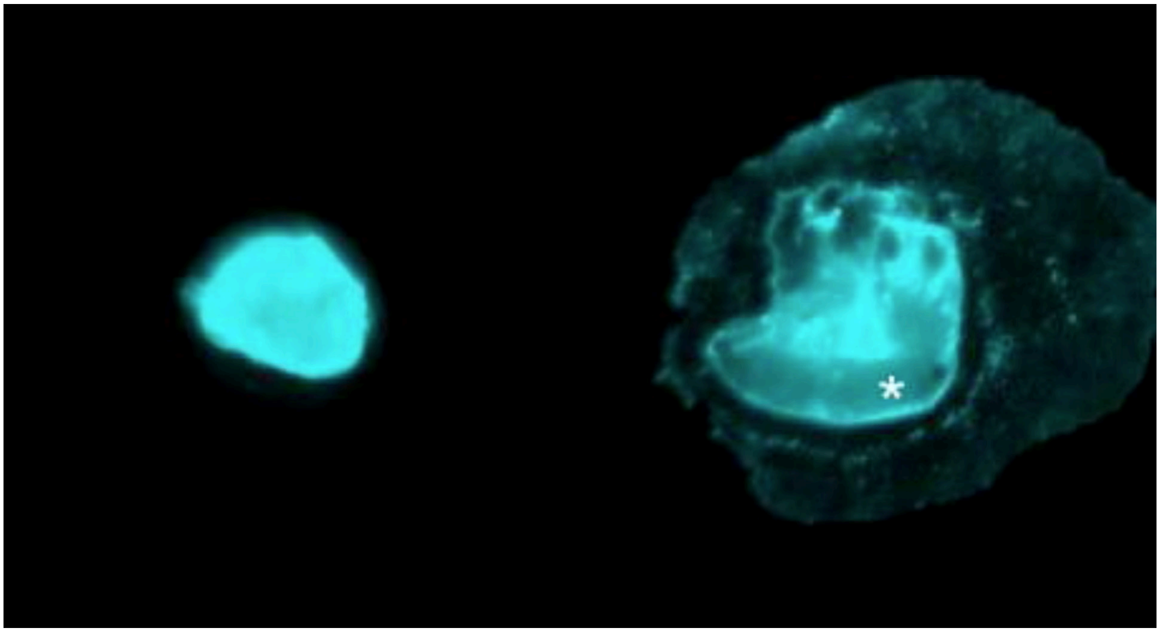

Supplementary Video 1: Movie of serial fluorescence images of tumor and small intestine after spraying Q_3STCy (left: tumor, right: small intestine). Asterisk indicate the small intestine.

See Supplementary Video 1

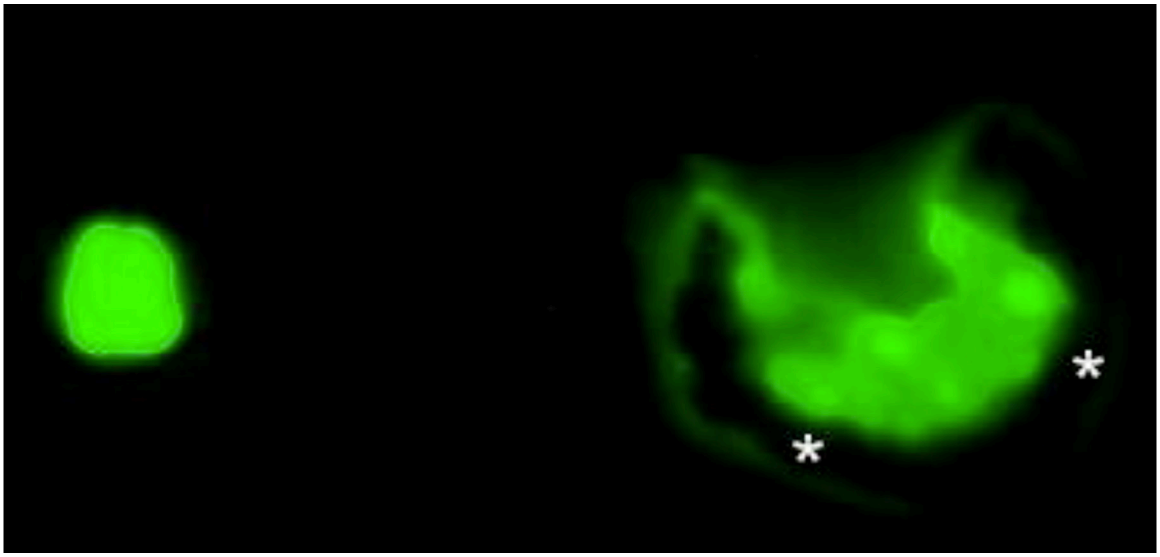

Supplementary Video 2: Movie of serial fluorescence images of tumor and small intestine after spraying gGlu-HMRG (left: tumor, right: small intestine). Asterisk indicate the small intestine.

See Supplementary Video 2
